# Ecological Sustainability: What Role for Public Health Education?

**DOI:** 10.3390/ijerph6072028

**Published:** 2009-07-23

**Authors:** Mary Louise Fleming, Thomas Tenkate, Trish Gould

**Affiliations:** School of Public Health, Queensland University of Technology, Victoria Park Road, Kelvin Grove, Queensland 4059, Australia; E-Mails: t.tenkate@qut.edu.au (T,T.); t.gould@qut.edu.au (T.G.)

**Keywords:** ecology, education, environment, public health, sustainability

## Abstract

This article explores the notion of ecological sustainability in the context of public health education and the contribution Universities can make in creating environments that include ecologically sustainable practices. It considers the important role of environmental health in building a sustainable future for the population as a central plank of public health. It presents the evidence for the need for comprehensive approaches to ecological sustainability within the University and offers suggestions about how this can take place. It concludes by arguing that to date there is a substantial gap between the rhetoric and the reality in the University context.

## 1. Introduction

There are a range of ways in which public health can contribute to sustainability; including the sustainability of the environment in which we live, the resources we use and the contribution we collectively and individually make to ecological sustainability. However, it is very important for us to remember that whatever contribution we allocate to environmental health or public health in the debate about sustainability, the fundamental issue is that without the contribution of many disciplines to ecological sustainability we will see limited success. One of the key issues that many governments are facing now is balancing the requirement to limit global changes, and their consequent impacts on ecological sustainability, with the social, political and economic problems that may result from imposing these limits. As Smyth [1] indicates “… our environment is the totality of what we live in, natural or constructed, spatial, social and temporal. It is an extension of ourselves, its health requiring the same care as our own health”. It is clear that our current way of life and reliance on resources are not sustainable. As McMichael [2] points out “our prevailing cultural values, technologies, and behaviours are not ecologically sustainable: on current trends the natural world cannot continue to furnish the ‘services’ upon which our societies, health, and lives depend”. He goes on to state: “Until the public health community highlights the centrality to the overall sustainability project of long-term population health, and particularly its dependence on maintaining Earth’s life-support systems, society will continue to miss the real point—namely, that ‘ecological sustainability’ is not just about maintaining the flows from the natural world that sustain the economic engine nor maintaining iconic species and iconic ecosystems. It is about maintaining the complex systems that support health and life. Population well-being and health, understood thus, become the real bottom line of sustainability” [2].

As early as 1972, at the Stockholm Conference on the Human Environment, the concept of sustainable development was being discussed – it was to mark a “significant paradigm shift in approaches to dealing with the environment and development, and heralded a major advance in thinking” [3]. In addition to that paradigm shift, “the value of reconnecting environmental and public health is related to the long-term benefits that will accrue. Proponents in both [the environmental health and public health] professions recognize that public health and environmental health are flip sides of the same coin. In truth, they are not separate fields at all. Public health *is* environmental health” [4]. For example, “Two of the greatest health-endangering correlates of urban environments and living are, first, overweight and obesity and, second, the increasing contribution of cities to greenhouse gas emissions and the attendant risks to safety, health and survival” [5].

Public health interventions can be informed in a variety of different ways by environmental health, ecology and health, and human ecology. The contribution of each of these elements to public health is not mutually exclusive, because approaches that deal with complex issues can also provide useful insights to deal with more basic issues. Environmental health, ecology and health, and human ecology can “be seen as complementary approaches to addressing overlapping problems in the areas of health, environment, and development” [6].

This paper highlights the extent to which there has been a convergence over the last 20 years in the work of each of these areas and strongly supports a further conceptual and methodological integration of knowledge and action to ensure comprehensive and sustainable environmental health gains. As Parkes *et al.* [6] quite rightly argue “research and applied programs that integrate biophysical and social sciences with public health practice can go some way toward addressing the deficiencies in each approach when taken on its own”.

The roles of social and economic development, as both drivers and mediators of hazardous environmental exposures, and the need for an ecologically sustainable development are increasingly important challenges in environmental health and in public health [6–8].

## 2. The Global Context for Change

Public health’s mission has long been to investigate and address the impacts of significant social and environmental change on the public’s health; however, now that the ecosystems that support us are so endangered, the sustainability and integrity of our planet must be considered synonymous with the sustainability of humans [9].

As long ago as the late 1980s the World Health Organization (WHO), through the *Ottawa Charter* [10], was advocating for the creation of supportive environments. This notion reinforces the contention that our societies are complex and interrelated and that health cannot and should not be separated from other goals. “The inextricable links between people and their environment constitute the basis for a socio-ecological approach to health” [10]. This idea of sustainable environments clearly suggests that we need to encourage reciprocal maintenance–to take care of each other, our communities and our natural environment. The WHO was arguing for an emphasis on the conservation of natural resources throughout the world as a global responsibility over 20 years ago [10]. In particular, the WHO argued for a systematic assessment of the health impact of a rapidly changing environment, –particularly in areas of technology, work, energy production and urbanization–as being essential to ensure positive benefit to the health of the public [10].

Despite these issues being raised by the WHO [10] there are still numerous examples of continuing changes to our environment that are likely to impact on our wellbeing. These include climate change, the decline in the ozone layer, the reduction in biodiversity, and alterations in the physical environment and ecological processes [3]. In addition to these global changes, the effects of climate change will, as the Intergovernmental Panel on Climate Change (IPCC) Third Assessment Report [11] clearly states, “fall disproportionately upon developing countries and people without the necessary economic resources, and thereby exacerbate inequities in health status and access to adequate food, clean water, and other resources”. This is echoed in the recent Human Impact Report from the Global Humanitarian Forum which concludes that the people who are “least responsible for greenhouse gas emissions are the world’s poorest communities who suffer most from climate change” [12]. This report further concludes that climate change currently results in over 300,000 deaths, seriously affects 325 million people and results in economic losses of US$125 billion. In addition, it estimates that over the next twenty years, the number of people affected by climate change will more than double, “making it the greatest emerging humanitarian challenge of our time” [12].

Links between health and economic development have been well documented for many years, but in more recent times they have become increasingly recognised, especially with respect to the relationship between health and poverty. Furthermore, the growing burden of both non-communicable and communicable diseases has become more evident. In the twenty first century, the world is facing the challenges associated with the re-emergence of infectious diseases, as well as the increasing burden of non-communicable diseases. Never before have we in public health faced the complexity associated with increases in infectious disease mortality, with growing mortality and morbidity from non-communicable diseases. During the past few decades, the health effects of development policies and practices have been insufficiently stressed, although the need for assessment of these effects has been highlighted. As von Schirnding [3] suggests, the opportunities and threats to health posed by globalisation have only recently been realised, with the implications of global interdependency highlighted by the threat of global climate change and the HIV/AIDS pandemic.

The United Nations Conference on Environment and Development (UNCED or ‘Earth Summit’) in 1992, in Rio de Janeiro [13] was a defining moment in recognizing the relationships between development and environmental issues. The ‘Earth Summit’ [13] resulted in the adoption of “Agenda 21, a wide-ranging blueprint for action to achieve sustainable development worldwide” [14]. Agenda 21 also acknowledged the significance of protecting and promoting human health [3]. More recently, the 2002 World Summit on Sustainable Development (WSSD) [15] yielded two documents: the first being the Political Declaration, which supports principles that were approved in 1992 at the ‘Earth Summit’ [13] and the Millennium Summit in 2000 [16]. This Declaration recognized that “poverty eradication, changing consumption and production patterns, and protecting and managing the natural resource base for economic and social development are overarching objectives of, and essential requirements for sustainable development” [15]. In addition, it identified that the ever-widening gap between rich and poor countries poses an increasing threat to global security, prosperity and stability, which, when combined with increasing adverse environmental change and global market forces, substantially threatens the long-term sustainability of the planet [15]. The second is the Plan of Implementation (the ‘action plan’), which outlines a number of recommendations on a range of issues, including water and sanitation, natural resources and biodiversity, and health [15]. The 2002 World Summit demonstrated clear relationships between sustainable development, human health and the environment [17].

Health has been given more prominence by the WHO for sustainable development, and is now one of the important concerns selected as necessitating particular consideration, together with biodiversity, water, agriculture and energy [3]. In addition, the WHO has chosen Climate Change and Health as its theme for 2008–09. McMichael [18] points out that the public health community needs to focus on efforts to undertake multi-sectoral actions that seek to create environmental and social conditions able to sustain population health. Probably “a quarter of the world’s total loss of healthy years of life is associated with environmental factors” [3]. In 1990, 23% of total DALY burden worldwide was caused by environmental factors [19]; and in 2004 it was 24% [20]. However, the real figures are probably higher, as there is limited data available for many diseases at present [20]. In addition, with the total number of healthy life years lost per capita due to the environmental burden (per capita) being 15 times higher in developing countries than developed, clearly, the environmental disease burden is unequally distributed [20].

The fundamental issue around global environmental change is that humans are collectively overloading the capacity of the Earth to supply, absorb, replenish, and stabilise, and this poses a profound, potentially irreversible, form of non-sustainability. McMichael [2] provides us with clear evidence from a number of examples about the impact of environmental change including the loss of species that cannot be regained; damage to our ecosystems (wetlands, forests, reefs, etc.) that cannot easily be rebuilt, if at all; the incredible variability of global climatic conditions; and the degradation and acidification of land, that even if possible, will take years to restore. Other authors have pointed out that excessive amounts of material and energy use and waste production jeopardize global sustainability, which inevitably results in delayed, indirect health effects [3,17]. “In between these extremes, many developing countries are experiencing the worst of both worlds: traditional risks associated with poverty; and new and emerging risks associated with large-scale, rapid industrialisation, urbanisation, and technological development” [3]. The impact of these changes extends “beyond the boundaries of cities and affect the population at large. For example, energy use in cities and the resultant greenhouse gas emissions have consequences, via climate change, for humans everywhere. The resultant health risks involve the impact of heatwaves, especially in cities; exacerbation of local air pollution; intensified extreme weather events; and heightened transmission of temperature-sensitive infections” [5].

It is clear that the challenges posed by global environmental change are substantial and require urgent and ongoing commitment and leadership at all levels of government and society. As the interconnectedness of the environmental, economic, political, social, and spiritual challenges is becoming increasingly obvious, it is also clear that there is a need for a “shared vision of basic values to provide an ethical foundation for the emerging world community” [21]. Such a vision is provided by the *Earth Charter* which provides sixteen “interdependent principles for a sustainable way of life as a common standard by which the conduct of all individuals, organizations, businesses, governments, and trans-national institutions is to be guided and assessed” [21]. From an educator’s perspective, Principle 14 emphasises the need to “integrate into formal education and life-long learning the knowledge, values, and skills needed for a sustainable way of life” [21]. Therefore, “from the outset, education has been at the center of the Earth Charter’s purpose and a major focus of the Earth Charter Initiative’s programmes” [22].

## 3. The Educational Agenda: An Important Role for Public Health

What is the role for universities through undergraduate and postgraduate education, and continuing professional practice education, particularly in the public health arena, in making a significant contribution to ecological sustainability?

Agenda 21 [14] unambiguously reaffirmed that education was critical for promoting sustainable development and improving the capacity of the people to address environment and development issues. Why is education so important? Both formal and non-formal education are indispensable in changing people’s attitudes so that they have the capacity to assess and address their sustainable development concerns. It is also critical for achieving environmental and ethical awareness, values and attitudes, skills and behaviour consistent with sustainable development, and for effective public participation in decision-making [14]. In addition, as universities are “an integral part of the global economy and since they prepare most of the professionals who develop, manage and teach in society’s public, private and non-government institutions, they are uniquely positioned to influence the direction we choose to take as a society” [23]. Therefore, universities have a critical and fundamental role (and some say a responsibility) for sustainability through their teaching and research activities [24,25]. The Association of University Leaders for a Sustainable Future state that “the success of higher education in the twenty-first century will be judged by our ability to put forward a bold agenda that makes sustainability and the environment a cornerstone of academic practice” [23].

Based on the way in which universities operate and the influence they have within the community, there would seem to be four interconnected elements that need to be addressed for them to achieve their role for sustainability: institutional policy and commitment, operational activities, teaching and research, and professional development/extension activities.

### 3.1. Institutional Policy and Commitment

It is clear that without a high level of commitment from the university executive (for example, the Chancellor, President) the effectiveness of any sustainability activities will be limited. One indicator of commitment by a university to sustainability is whether they have signed the *Talloires Declaration*. Composed in 1990, this was the first official statement made by university executives of a commitment to ecological sustainability in higher education. It is a ten-point action plan for incorporating sustainability and environmental literacy into all aspects of university operations. Currently, 407 higher education institutions from over 50 countries have signed this Declaration [23]. However, there is no monitoring of the *Talloires* signatories and there is no enforcement mechanism to ensure that they are following-through on their commitments [26]. In fact, a number of reports have indicated that universities who have adopted this or other sustainability policies or declarations often fail to implement their basic commitments [27–30]. Often, there are ‘pockets’ of commitment and activity which are *ad hoc* and at a ‘grassroots level’, whereas full integration and transformation of university practice is rare [26,31]. Some key reasons for this include a lack of commitment from executive management and a lack of acceptance from staff [30], and a lack of accountability for not delivering on these commitments [31]. As such, Bekessy *et al.* [31] propose that the following two elements are a sign of a university which is serious in following through on its commitments: (1) an appropriate long-term budget is assigned to ensure that resources are available, because without this, any activities undertaken are likely to be inadequate to achieve genuine transformative change; and (2) the university’s progress towards sustainability should be exposed to the scrutiny of the international public, academic and corporate arena.

### 3.2. Operational Activities

As university campuses are like small cities, their impacts can be substantial. In addition to being large employers, their decisions on energy, waste and water use, or whether their purchasing decisions take sustainability into account, can all have a significant impact. Consequently, it is vitally important that universities take sustainability seriously in daily operations (e.g., materials purchasing, energy and water use, waste management) and in campus management (e.g., buildings, outdoor ‘green spaces’), so as to reduce their ecological impacts and to provide good examples to students and the community [26]. In this regard, a number of surveys have found that universities are undertaking substantial sustainability initiatives and are increasingly using sustainability principles when purchasing new equipment or products, and when constructing or renovating buildings [32,33]. Accordingly, the ‘greening of campuses’ approach is one aspect of sustainability education that universities seem to have embraced, however, it is dominated by project-based operational activities, with linkages to research and teaching rarely achieved [34].

### 3.3. Teaching and Research

There are two ways in which sustainability can be incorporated into university teaching: one is to ensure that all degree programs produce sustainability-literate graduates, and the other is to produce some sustainability specialists [25]. There are many advocates for sustainability to become a theme that transcends and encompasses all disciplines [26]. In fact, Soskolne [35] states that “in our view, it is a disservice to permit any student in any discipline to emerge from an [tertiary] education without a deeper understanding of sustainability”. While some universities mandate courses on sustainability as a requirement for graduation for all students [36], the integration of sustainability into mainstream curriculum is not highly developed [25,37], despite the international calls for this to occur (particularly through the UN’s Decade of Education for Sustainable Development [38]).

In particular, the education of public health practitioners “must look beyond the biological risk factors that affect health and seek to also understand the impact on health of environmental, social, and behavioral factors” [39]. The effectiveness of interventions will undoubtedly be influenced by the interaction of multiple factors. An understanding of the theoretical underpinnings of the ecological model will be essential in order to develop research that further explicates the pathways and interrelationships of the multiple determinants of health. The importance of a good quality education will be paramount to developing this understanding for public health professionals and will enable them to more successfully tackle contemporary issues, such as demographic upheavals, scientific and medical technologies, and globalization [39]. Brown *et al.* [9] draw on a wide range of sources to support the notion that in view of the latest reports on the state of the environment, it is vital that the public health profession reviews its responsibility in protecting human health.

Griffith [40] states that it is important to integrate the ecological sustainability paradigm into all the public health-related disciplines, rather than merely encouraging inter-sectoral collaboration and partnerships; such as between the disciplines of environmental health and public health. Furthermore, ecological sustainability must consistently be a part of the blueprint of all public health initiatives and interventions; specifically, all such programs must include relevant procedures to safeguard and enhance the natural and built environment [40]. A range of suggestions for embedding the concept of ‘ecological sustainability’ in public health have been posed [40,41], including the following, which are pertinent to public health *education*:

- We need to realize that the domain of environmental health incorporates ecological and global concerns, since those concerns influence the future of public health [41].
- Competences on ecological sustainability could be included in National Occupational Standards, and in the competency frameworks, standards and curricula of all the public health professions and disciplines [40]. “This goal must be accomplished through formal education, continuing education, and distance learning” [41].
- Ecological sustainability could be included in public health leadership programmes, and in public health teaching networks linking educational institutions and service departments [40]. We should ensure that basic environmental health competencies are inculcated into present and future environmental health and protection workforces [41].
- Ecological sustainability could be included routinely in public health training programmes [40]. We should also recognize that environmental health remains a basic component of the field of public health, no matter what or how agencies organize and deliver services [41].
- Appropriate responsibilities in relation to ecological sustainability could be included in job descriptions across all the professions and disciplines that contribute to public health [40]. Regardless of the educational disciplines and occupations concerned, such graduates should be taught public health practice [41].
- Schools of Public Health need to employ “academically qualified environmental health faculty” with relevant work experience, who “will serve as practitioner role models and mentors” [41].
- We should regularly develop and utilize links between “all the interests involved in environmental health and protection issues. These interests include engineers, architects, land use and transportation planners, public works organizers, conservationists, economic development officials, agricultural interests, resource developers, the medical community, housing interests, and environmental advocacy groups” [41].

In addition, education curricula must be relevant to the requirements of particular “goal-oriented research” and must endeavour to turn out specialists knowledgeable about ecologically ‘sound’ technology and incorporate an inter-sectoral approach [42]. McMichael’s [2] comments clearly suggest that health researchers have been slow to engage with the notion of ecological sustainability, and he offers suggestions about why this is the case: “First, it reflects the inherent conservatism of science in general; a reluctance to look beyond defined professional boundaries and paradigms. Second, more generously, the slowness also reflects the enormous quantum-like leap that is required, from studying specific, local, mostly direct-acting ‘exposures’ to studying how changes in whole natural systems can, via varied pathways and over protracted time, affect health” [2].

We would argue therefore that public health curricula should focus strongly on the concept of ‘ecological public health’ as it has “evolved in response to the changing nature of health issues and their interface with emerging global environmental problems. These new problems include global ecological risks such as the destruction of the ozone layer, uncontrolled and unmanageable air and water pollution, and global warming. These developments have a substantial impact on health which often elude simple models of causality and intervention” [43]. As such, “ecological public health emphasises the common ground between achieving *health* and *sustainable development*. It focuses on the economic and environmental *determinants of health*, and on the means by which economic investment should be guided towards producing the best population *health outcomes*, greater *equity in health*, and sustainable use of resources” [43]. These concepts can also be considered to be fundamental sustainability literacies for all graduates, and are emphasised through the *Earth Charter* [22].

### 3.4. Professional Development/Extension Activities

Universities are well placed to provide professional development for practitioners as well as to undertake outreach/extension activities in the community, particularly through developing partnerships with schools, government, non-governmental organizations and industry. They should also support research partnerships that fulfil community objectives for sustainability across a wide range of disciplines [27]. Despite some examples of universities undertaking extension projects which are designed to empower and educate community members [44]; professional development activities for the community or to introduce their own academics to sustainability, and the teaching of sustainability, appear to be limited [37,45]. Thus, considerable professional development of academic staff is needed to help them appreciate how they can lead the next generation to global sustainability [45].

No matter what the mode of delivery, the discipline studied, or the setting through which the training is undertaken, universities should strive to ensure that they help students, staff and the community to [22]:

- Understand the challenges and critical choices that humanity faces and appreciate the interconnections between these challenges and choices;
- Comprehend the meaning of a sustainable way of life and of sustainable development, and to create personal goals and values which are conducive to a sustainable way of life; and
- Critically evaluate a given situation and identify action goals to bring about positive change.

For public health students and practitioners, these fundamental literacies are critical because environmental problems and health are becoming progressively more complicated, inter-sectoral and interconnected [2,3,5,46], and public health is becoming acknowledged more and more as a pivotal factor in sustainable development, and is a fundamental policy issue central to “a country’s economic development” [3].

## 4. Conclusions

As Parks *et al.* state, “recent trends in environmental health, ecology and health, and human ecology all suggest that the interface between sustainability, ecosystems, social systems, and health is fertile ground for optimizing environmental health interventions and maximizing public health gain” [6]. With an accelerated rate of economic development, the substantial increase in the world population and the globalisation of trade, these modern realities have dramatically changed production methods and demand for goods in both developed and developing countries, and have become contemporary challenges for disciplines like public health and environmental health. These changes in the way we live and the ever-increasing impacts of human activity on environmental resources and systems highlight an ever-increasing urgency for all to understand that population health is an important part of the concept of sustainability [5]. Accomplishing sustainable social, economic and environmental conditions therefore underpins the achievement of population health [47]. Further, documents such as the *Earth Charter* argue for the integration into formal education and more broadly into life-long learning the knowledge, values, and skills needed for sustainable living [21]. In this context, universities play a critical role in embedding sustainability principles and understanding in society, through the training of future leaders and professionals, cutting-edge research, and community outreach activities that empower local communities to implement sustainable principles and practices. However, universities currently have a mixed track record when it comes to sustainability–they have embraced various ‘greening of campuses’ approaches, but there seems to be limited transformation of genuine university practice, particularly due to a lack of high-level commitment with little accountability, as well as minimal integration of sustainability into mainstream curriculum.

This article has argued that the development of a workforce that recognises and responds to local agendas, as well as recognising that we are now players on the global stage, is only one component of the necessary actions for ecological sustainability. The University sector needs to be committed to the concept of ecological sustainability and put that concept into practice. Ecological sustainability is our biggest challenge – as educators, as practitioners and as citizens. The future poses its challenges and if those challenges are met the sustainability of our total environment will be our reward.
